# Betamethasone latency period and neonatal hypoglycemia in term infants

**DOI:** 10.1080/14767058.2025.2540477

**Published:** 2025-08-03

**Authors:** Lauren A. Buckley, Briana Clifton, Katelyn M. Tessier, Connor Demorest, Raghavendra B. Rao, Sarah A. Wernimont

**Affiliations:** a Department of Pediatrics, Division of Neonatology, University of Minnesota, Minneapolis, MN, USA; b Department of Obstetrics, Gynecology and Women’s Health, University of Minnesota, Minneapolis, MN, USA; c Masonic Cancer Center Biostatistics Core, University of Minnesota, Minneapolis, MN, USA

**Keywords:** Antenatal, betamethasone, corticosteroid, glucose, hypoglycemia

## Abstract

**Introduction:**

Infants exposed to antenatal betamethasone (BMZ) between 34.0 and 36.6 weeks gestational age (GA) have increased risk for neonatal hypoglycemia, a known cause of brain injury. While a shorter latency period between BMZ administration and delivery is associated with an increased risk of neonatal hypoglycemia in late preterm infants, the impact of the BMZ latency period on neonatal hypoglycemia in infants born at term (≥37 weeks) has not been previously characterized. Term infants without additional risk factors such as growth restriction or maternal diabetes are not routinely screened and may be at risk for unrecognized neonatal hypoglycemia. The purpose of this study was to determine whether the latency period from antenatal BMZ administration to delivery impacts the incidence of neonatal hypoglycemia in term infants.

**Methods:**

This retrospective cohort study analyzed maternal–infant dyads from the University of Minnesota Obstetric Measures database (January 2017–August 2023) who received BMZ during pregnancy and delivered at ≥37 weeks (*N* = 758). The primary outcome was incidence of early neonatal hypoglycemia, defined as a blood glucose level <40 mg/dL within 48 h of birth, in neonates exposed to BMZ <14 days (recent BMZ; *N* = 161) vs. >14 days (remote BMZ; *N* = 597) prior to delivery. Secondary outcomes included incidence of severe neonatal hypoglycemia (blood glucose level <25 mg/dL within 48 h of birth) and treatment for neonatal hypoglycemia with dextrose gel or IV dextrose. Demographics, pregnancy characteristics, neonatal characteristics, and outcomes were summarized by time from last dose of BMZ to delivery.

**Results:**

The most common indication for BMZ was pre-eclampsia/pregnancy-induced hypertension in the recent BMZ group and preterm labor with intact membranes in the remote BMZ group (*p* < .001). Maternal diabetes status did not differ between groups. The recent BMZ group had lower median GA at birth (37.1 vs. 38.4 weeks, *p* < .001) and lower birth weight (3.03 vs. 3.29 kg, *p* < .001). The percentage of neonates classified as small for GA or large for GA was similar between groups. Approximately 30% of the neonates had a documented glucose measurement within 48 h of birth. The incidence of early neonatal hypoglycemia, severe hypoglycemia, and treatment for hypoglycemia with dextrose gel or IV dextrose did not differ between infants exposed to recent BMZ vs. those exposed to remote BMZ.

**Discussion:**

In this cohort of term infants, a shorter latency period between BMZ administration and delivery was not associated with increased incidence or treatment of hypoglycemia. However, the rate of hypoglycemia screening was low in both groups and rates of undiagnosed hypoglycemia in BMZ-exposed term neonates remain unknown. Prospective longitudinal studies aimed at characterizing the risk of hypoglycemia in term infants exposed to BMZ would be beneficial to inform hypoglycemia screening practices in BMZ-exposed neonates.

## Introduction

Administration of antenatal betamethasone (BMZ) to pregnant individuals with threatened preterm birth is a well-established and proven therapy for reducing morbidity and mortality in preterm infants [1]. Accurately predicting preterm births is challenging, and up to 40% of infants exposed to BMZ for threatened preterm birth are ultimately born at term [2]. While the benefits of BMZ are undisputed for preterm infants, term infants see no therapeutic benefit and may experience harm related to antenatal BMZ exposure [3]. BMZ exposure in term infants has been linked to impaired cortisol-reactivity, behavioral and psychiatric problems in childhood [3–8] and hypoglycemia in the neonatal period [9], raising concerns about underrecognized consequences of this widespread therapy.

One possible consequence of BMZ exposure, neonatal hypoglycemia, is a known cause of brain injury in newborns [10–13]. The American Academy of Pediatrics recommends routine screening for neonatal hypoglycemia in infants with certain risk factors including late preterm birth, gestational diabetes, and small or large for gestational age (SGA or LGA) status [10–15]. In 2016, the antenatal late preterm steroids (ALPSs) trial demonstrated a link between BMZ administered during the late preterm period (34–36 weeks of gestation) and increased risk for neonatal hypoglycemia [16–18], with follow-up studies indicating that the risk of hypoglycemia was highest for infants with a short latency period between BMZ administration and delivery. While hypoglycemia hours-to-days after BMZ exposure is attributed to maternal hyperglycemia and transient hyperinsulinism in the newborn, it is unknown whether glucocorticoid-mediated programming of the developing hypothalamic-pituitary-adrenal axis and fetal metabolic tissues may impact neonatal glucose homeostasis even when BMZ exposure occurs well before delivery [3,16,17,19,20]. Routine screening for neonatal hypoglycemia in steroid-exposed infants is not presently recommended, and variables mediating the risk of neonatal hypoglycemia in term infants exposed to BMZ have not been well defined.

The objective of this study was to determine whether the latency period from BMZ administration to delivery impacts the incidence of neonatal hypoglycemia and hypoglycemia-related interventions in term infants. We hypothesized that term infants with recent BMZ exposure (<14 days prior to delivery) would have higher incidence of neonatal hypoglycemia than term infants with remote BMZ exposure (>14 days prior to delivery).

## Materials and methods

This is a retrospective cohort study of maternal–infant dyads who received BMZ due to perceived risk for preterm birth, yet were delivered at term gestational age (GA).

### Study participants

The study population was derived from the University of Minnesota Obstetric Measures database (Wernimont SA, Principal Investigator [IRB #STUDY00012822]), which is an obstetric outcomes database including >115,000 births from 2011 to 2023 within the M Health Fairview system [21]. M Health Fairview is an academic-community health system partnership that includes a large tertiary care academic hospital and multiple satellite hospitals, including a total of eight delivery centers spanning urban, suburban, and rural settings throughout the State of Minnesota. This study was reviewed by the Institutional Review Board at the University of Minnesota and determined to be exempt (IRB #STUDY00019336).

Eligible maternal–infant dyads included singleton pregnancies delivered at ≥37 weeks GA between January 2017 and August 2023, who received at least one dose of BMZ during pregnancy. We limited inclusion to deliveries from 2017 onwards to capture a sample representative of contemporary BMZ prescribing practices, which were liberalized to include those at risk for late preterm delivery after the ALPS trial was published in April 2016 [18,22]. To ensure that maternal data were not duplicated within the cohort, we excluded multifetal gestations, and in cases of subsequent qualifying births to the same individual, we included only the most recent delivery.

### Study outcomes

The primary outcome was neonatal hypoglycemia (blood glucose <40 mg/dL within 48 h of delivery). Secondary outcomes were severe hypoglycemia (blood glucose <25 mg/dL within 48 h of delivery), treatment of neonatal hypoglycemia with dextrose gel or 10% dextrose (D10) bolus, and hospital length of stay (LOS). Thresholds for hypoglycemia and severe hypoglycemia were chosen in accordance with the neonatal hypoglycemia treatment algorithm published by the American Academy of Pediatrics Committee on the Fetus and Newborn [15]. Recent BMZ was defined as administration of the last dose <14 days prior to delivery and remote BMZ was defined as administration of the last dose >14 days prior to delivery.

### Data collection

Timing of BMZ administration was determined based on documentation in the medical administration record. Demographic data were abstracted from the electronic medical record. Diabetes status, hypertensive disorders of pregnancy, and indication for antenatal steroids were determined by manual review of the electronic medical record. Type 1 and type 2 diabetes were classified based on pre-pregnancy diagnoses. Gestational diabetes was defined based on American College of Obstetrics and Gynecology criteria using the two-step testing method [23]. Hypertensive disorders of pregnancy were classified in accordance with American College of Obstetrics and Gynecology guidelines [24].

SGA and LGA status were defined as birth weight <3rd or >90th percentile, respectively, for GA using the Fenton growth chart [25]. Culture positive sepsis was defined as a positive blood, urine, or cerebral spinal fluid culture. Polycythemia was defined as hematocrit >65% or hemoglobin >22 g/dL. Need for any respiratory support >6 h, mechanical ventilation and therapeutic hypothermia were determined by manual chart review of infants who were admitted to the neonatal intensive care unit (NICU). All neonatal characteristics were inclusive of the timeframe from birth to hospital discharge.

Glucose measurement within 48 h was defined as the existence of any plasma or whole blood glucose level documented in the electronic medical record from birth to 48 h of life. Hypoglycemia and severe hypoglycemia were defined as the existence of any plasma or blood glucose level less than 40 mg/dL or 25 mg/dL, respectively, anytime from birth to 48 h of life. Any medical intervention for hypoglycemia within 48 h was defined as dextrose gel application to the buccal mucosa or administration of a D10 bolus (200 mg/kg) IV from birth to 48 h of life.

### Power calculation

We estimated the incidence of neonatal hypoglycemia to be 15% and 24% in baseline and high-risk populations, respectively [18,26]. Assuming a 1:3 enrollment ratio with a significance level of 5% (*a* = 0.05) and 80% power (*b* = 0.8), we calculated *a priori* that a sample size of at least 640 would be required to detect a 10% difference in incidence of hypoglycemia between groups [27].

### Statistical methods

Maternal demographics, pregnancy characteristics, neonatal characteristics, and outcomes were summarized by time from last dose of BMZ to delivery. To investigate the association between exposure and continuous variables, Student’s *t*-tests or Wilcoxon’s rank-sum tests were used. Chi-square or Fisher’s exact tests were used for categorical variables. Unadjusted and adjusted (for diabetes, hypertension, birthweight category, and indication for antenatal steroids) logistic regression models were used to investigate the effect of BMZ latency period on neonatal glucose outcomes. Odds ratios (ORs) and 95% confidence intervals (CI) were obtained. All reported *p* values are two-sided and a significance level of .05 was used. Statistical analyses were performed using R (version 4.1.2, R Core Team, R Foundation for Statistical Computing, Vienna, Austria).

## Results

**Figure 1.**
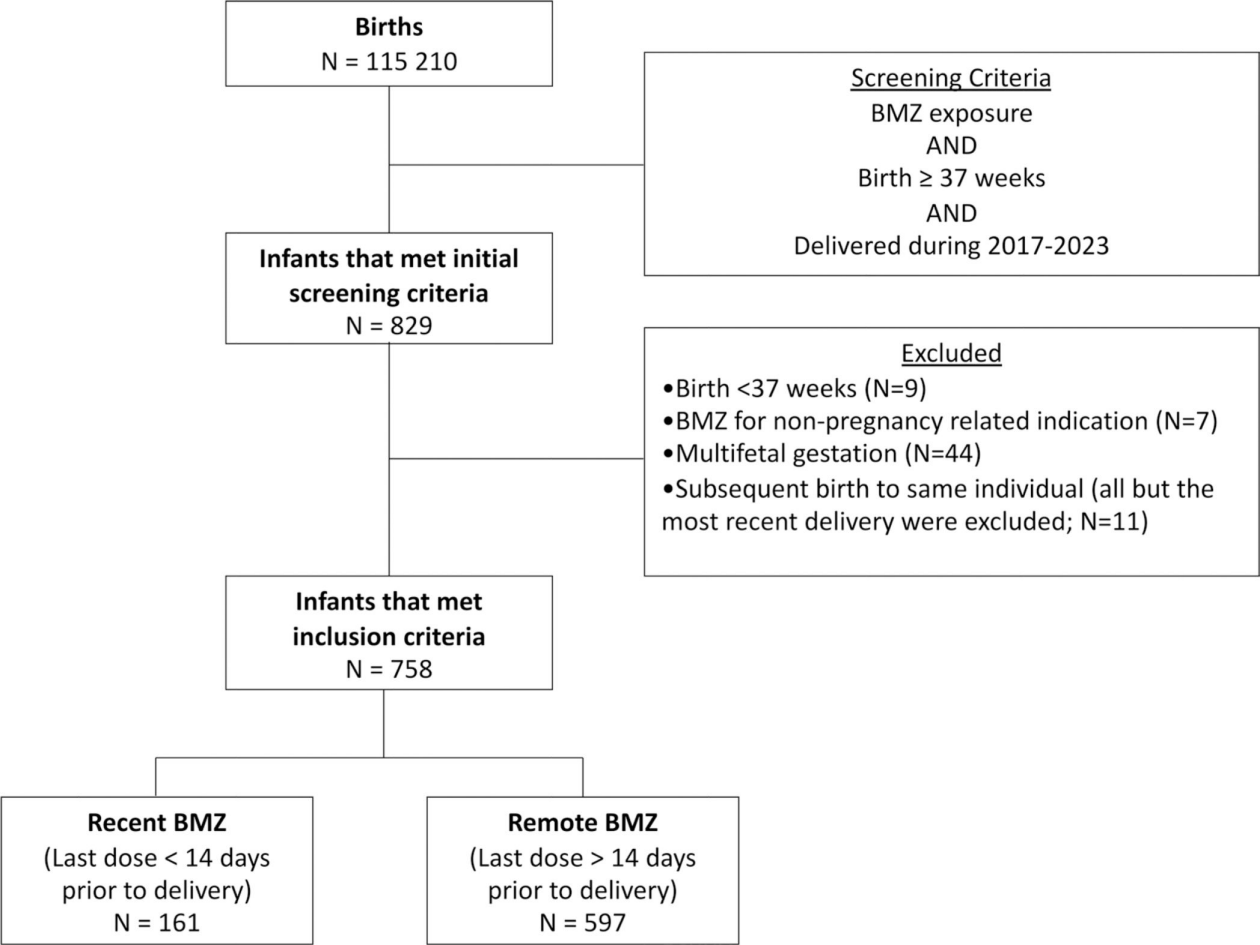
Flow diagram of infants meeting study inclusion criteria. BMZ: betamethasone.

There were 115,210 births in the University of Minnesota Obstetric Measures database from January 2011 to August 2023 (Figure 1). Of these, 829 births met the initial screening criteria of BMZ exposure, birth ≥37 weeks GA and delivery during 2017–2023. After manual chart review, a total of 60 infants were excluded for birth GA <37 weeks (*N* = 9), BMZ administration for a non-pregnancy related indication such as a joint injection (*N* = 7), or multifetal gestation (*N* = 44). To prevent duplicated maternal data within the study sample, an additional 11 births were excluded due to the same individual having a subsequent birth that met inclusion criteria (all but the most recent birth were excluded). The remaining 758 infants were divided in two groups based on timing of BMZ relative to delivery: *N* = 161 infants in the recent BMZ group (<14 days prior to delivery) and *N* = 597 infants in the remote BMZ group (>14 days prior to delivery).

There were several significant differences in maternal demographics, pregnancy characteristics, and BMZ administration between groups (Table 1). While mean maternal age was higher in the recent BMZ group (32.3 vs. 30.9 years, *p* = .005), maternal race, insurance, parity, and mode of delivery did not differ between groups. Hypertensive disorders in pregnancy were more common in the recent BMZ group (55.3% vs. 27.0%, *p* < .001). There was no difference in maternal diabetes status. Indication for antenatal BMZ also differed; pre-eclampsia or pregnancy-induced hypertension was the most common indication in the recent BMZ group, while preterm labor with intact membranes was most common in the remote BMZ group (*p* < .001). GA at first dose of BMZ was higher in the recent BMZ group (*p* < .001), with 96.3% of infants receiving their first dose in the late preterm period (≥34 weeks), compared with 31.2% of infants in the remote BMZ group receiving their first dose during late preterm gestation.

Infants in the recent BMZ group were born at an earlier GA (median 37.1 days vs. 38.4 days, *p* < .001) and correspondingly had lower birth weight (mean 3.03 kg vs. 3.28 kg, *p* < .001) and longer hospital LOS (median 2.1 vs. 1.9 days, *p* = .008) compared to infants in the remote BMZ group (Table 2). There were no differences in infant sex, proportion of SGA and LGA infants, Apgar scores, NICU admission, culture positive sepsis, polycythemia, phototherapy, respiratory support >6 h, mechanical ventilation, or therapeutic hypothermia.

There were no significant differences in the incidence of hypoglycemia and related outcomes between infants with recent BMZ vs. remote BMZ exposure (Table 3). Approximately, 30% of the neonates had at least one documented glucose measurement within 48 h of delivery and there was no difference in the likelihood of having a documented glucose measurement between the two groups. When compared with infants with remote BMZ exposure, infants with recent BMZ exposure were not more likely to have hypoglycemia, severe hypoglycemia, any medical intervention for hypoglycemia within 48 h, dextrose gel within 48 h, or D10 bolus within 48 h of delivery. After adjustment for maternal diabetes, maternal hypertension, birthweight category, and indication for antenatal steroids, there remained no significant difference between groups.

Finally, a post hoc analysis was completed to test the hypothesis that BMZ exposure only impacts hypoglycemia risk in term infants if administered within a shorter interval to delivery (<3 days). Here, we compared hypoglycemia related outcomes in infants who received BMZ <3 days prior to delivery (*N* = 37) with infants who received BMZ >14 days prior to delivery (*N* = 597) and again found no difference in any of the hypoglycemia related outcomes between groups (Supplemental Table 1).

## Discussion

In this cohort of term infants exposed to antenatal BMZ, the timing of BMZ exposure relative to delivery did not impact the incidence of hypoglycemia, severe hypoglycemia, or treatment for hypoglycemia during 48 h after birth. Similar results were obtained on post hoc analysis comparing only the subset infants with the shortest BMZ latency interval (<3 days prior to delivery) to infants with remote BMZ exposure (>14 days). These results are contrary to our hypothesis that infants with more recent BMZ exposure would have increased incidence of neonatal hypoglycemia. The findings are notable in light of the fact that the recent BMZ group had lower GA at delivery and lower birth weight, two factors that are generally associated with increased risk for neonatal hypoglycemia.

Relatively few studies have examined the effect of latency period from BMZ administration to delivery on the incidence of neonatal hypoglycemia. McElwee et al. previously demonstrated that a latency period of 12–71 h is associated with highest risk of neonatal hypoglycemia in infants exposed to antenatal BMZ at late preterm gestation [28]. Similarly, a secondary analysis of the ALPS cohort showed the highest association between BMZ exposure and elevated insulin and leptin levels in cord blood of late preterm infants exposed BMZ between 12 and 24 h prior to delivery [16]. Finally, di Pasquo et al. reported a shorter latency period from steroid administration to birth (1.8 vs. 3.3 weeks) in hypoglycemic vs. normoglycemic infants exposed to antenatal corticosteroids, though this relationship was no longer significant on multivariate regression analysis for predictors of neonatal hypoglycemia [29]. Our study differs from these in that it included infants with a much longer interval between BMZ administration and delivery. Despite these differences in methodology, our results suggest that previously reported findings by McElwee, Battarbee, and di Pasquo may not be generalizable to infants with BMZ exposure who are born at term.

Our study has several strengths. First, these data come from a large academic-community outcomes database and are therefore able to capture maternal–infant dyads in a variety of care settings including academic, suburban, and rural centers, resulting in a relatively large and diverse study sample. Additionally, clinical diagnoses were validated by manual chart review for all maternal subjects and all infants requiring NICU admission, minimizing the risk for missing or inaccurate data from automated searching of electronic medical records using ICD-10 codes. There are also several important limitations to our study. First, this is a retrospective study in a patient population that is not routinely screened for neonatal hypoglycemia; therefore, only about a third of the study participants had any documented glucose level. When the comparison is limited to only those infants with a documented glucose measurement, the rate of hypoglycemia remains similar between infants with recent vs. remote BMZ exposure (35% vs. 41% respectively, *p* = .433, *N* = 224). Nevertheless, the incidence of undetected neonatal hypoglycemia is unknown and may vary between groups, impacting results. Given the retrospective nature of this study, it was not possible to fully adjust for unknown confounding variables. Among the patients who were screened for hypoglycemia, there is potential for selection bias since an infant with risk factors or borderline glucose measurements is likely to have more frequent blood glucose monitoring. Finally, we defined hypoglycemia as blood glucose <40 mg/dL for all infants <48 h of age. There is lack of consensus among experts regarding the precise definition of neonatal hypoglycemia, and we did not account for the fact that the glucose threshold at which injury occurs is likely lower in the initial hours after birth [15].

In this cohort of term infants, a shorter latency period between BMZ administration and birth was not associated with increased detection or treatment of neonatal hypoglycemia. However, the rate of hypoglycemia screening was low in both groups and the incidence of undiagnosed hypoglycemia in BMZ-exposed term infants remains unknown. Since fetal programming effects can have implications on human health across the lifespan, prospective longitudinal studies of term infants exposed to antenatal BMZ will be helpful to better define metabolic and neurologic outcomes in this patient population.

## Supplementary Material

Supplemental Table 1

Supplemental data for this article can be accessed online at https://doi.org/10.1080/14767058.2025.2540477

## Figures and Tables

**Table 1.** Comparison of demographics and pregnancy characteristics by time from last BMZ dose to delivery.

Variable	Recent BMZ^a^ (*N* = 161)	Remote BMZ^b^ (*N* = 597)	*p* Value^c^

Age (years), mean (SD)	32.3 (5.3)	30.9 (5.6)	.005
Race^d^, *n* (%)			.136
White	127 (84.7)	424 (75.3)	
Black	7 (4.7)	54 (9.6)	
Asian	11 (7.3)	65 (11.5)	
American Indian or Alaskan Native	3 (2.0)	7 (1.2)	
Native Hawaiian or Pacific Islander	0 (0.0)	1 (0.2)	
More than 1 race	2 (1.3)	12 (2.1)	
Insurance type, *n* (%)			.093
Uninsured	4 (2.5)	24 (4.0)	
Public	50 (31.1)	232 (38.9)	
Private	107 (66.5)	341 (57.1)	
Nulliparous, *n* (%)	50 (31.1)	211 (35.3)	.310
Diabetes, *n* (%)			.768
Type 1	0 (0.0)	4 (0.7)	
Type 2	0 (0.0)	6 (1.0)	
Gestational A1	7 (4.3)	30 (5.0)	
Gestational A2	5 (3.1)	23 (3.9)	
Hypertensive disorder in pregnancy, *n* (%)			<.001
Pre-eclampsia	42 (26.1)	58 (9.7)	
Gestational hypertension	40 (24.8)	73 (12.2)	
Chronic hypertension	7 (4.3)	30 (5.0)	
Indication for antenatal steroids, *n* (%)			<.001
Preterm premature rupture of membranes	0 (0.0)	15 (2.5)	
Preterm labor with intact membranes	46 (28.6)	363 (60.8)	
Pre-eclampsia or pregnancy-induced hypertension	79 (49.1)	87 (14.6)	
Fetal growth restriction	9 (5.6)	13 (2.2)	
Oligohydramnios	2 (1.2)	4 (0.7)	
Vaginal bleeding, abruption, or hemorrhage	8 (5.0)	66 (11.1)	
Other	17 (10.6)	49 (8.2)	
GA at 1st dose of BMZ, *n* (%)			<.001
≤27^6^ weeks	4 (2.5)	63 (10.6)	
28^0^ to 33^6^ weeks	2 (1.2)	348 (58.3)	
≥34^0^ weeks	155 (96.3)	186 (31.2)	
Number of doses of BMZ received, median [IQR]	2.0 [2.0,2.0]	2.0 [2.0,2.0]	.600
Mode of delivery, *n* (%)			.306
Vaginal	97 (60.2)	394 (66.0)	
C-section	59 (36.6)	181 (30.3)	
Assisted	5 (3.1)	22 (3.7)	

Abbreviations: BMZ, betamethasone; GA, gestational age; SD, standard deviation; IQR, interquartile range; gestational A1, managed with diet/exercise; gestational A2, managed with medication.

a Recent BMZ defined as last dose <14 days prior to delivery.

b Remote BMZ defined as last dose >14 days prior to delivery.

c *p* Value is for Student’s *t*-test or Wilcoxon’s rank-sum test for continuous variables, and Chi-square or Fisher’s exact test for categorical variables.

d Missing race for 45 individuals.

**Table 2.** Comparison of neonatal characteristics by time from last BMZ dose to delivery.

Variable	Recent BMZ^a^ (*N* = 161)	Remote BMZ^b^ (*N* = 597)	*p* Value^c^

GA at birth (weeks), median [IQR]	37.1 [37.0, 37.4]	38.4 [37.4, 39.1]	<.001
Infant sex, *n* (%)			.572
Female	79 (49.1)	278 (46.6)	
Male	82 (50.9)	319 (53.4)	
Birth weight^d^ (kg), mean (SD)	3.02 (0.46)	3.28 (0.47)	<.001
Birth weight category^d^, *n* (%)			.756
SGA	15 (9.4)	49 (8.3)	
AGA	131 (81.9)	479 (81.2)	
LGA	14 (8.8)	62 (10.5)	
Apgar score 1 minute^e^, median [IQR]	8.0 [8.0,9.0]	8.0 [8.0,9.0]	.793
Apgar score 5 minute^e^, median [IQR]	9.0 [9.0,9.0]	9.0 [9.0,9.0]	.335
NICU admission, *n* (%)	16 (9.9)	57 (9.5)	.882
Hospital LOS (days), median [IQR]	2.1 [1.6, 2.9]	1.9 [1.5, 2.5]	.008
Culture positive sepsis, *n* (%)	1 (0.6)	1 (0.2)	.380
Polycythemia, *n* (%)	1 (0.6)	0 (0.0)	.212
Phototherapy, *n* (%)	13 (8.1)	41 (6.9)	.597
Any respiratory support >6 h, *n* (%)	8 (5.0)	21 (3.5)	.394
Mechanical ventilation, *n* (%)	3 (1.9)	7 (1.2)	.450
Therapeutic hypothermia, *n* (%)	0 (0.0)	2 (0.3)	>.999

Abbreviations: BMZ, betamethasone; GA, gestational age; IQR, interquartile range; SD, standard deviation; SGA, small for gestational age; AGA, appropriate for gestational age; LGA, large for gestational age; LOS, length of stay.

a Recent BMZ defined as last dose <14 days prior to delivery.

b Remote BMZ defined as last dose >14 days prior to delivery.

c *p* Value is for Student’s *t*-test or Wilcoxon’s rank-sum test for continuous variables, and Chi-square or Fisher’s exact test for categorical variables.

d Birth weight data missing for 8 infants.

e Apgar score data missing for 6 infants.

**Table 3.** Comparison of glucose and hypoglycemia outcomes by time from last BMZ dose to delivery.

			Unadjusted model^a^		Adjusted model^b^	
Variable	Recent BMZ^c^ (*N* = 161)	Remote BMZ^d^ (*N* = 597)	OR (95% CI)	*p* Value	OR (95% CI)	*p* Value

Any glucose measurement within 48 h, *n* (%)	54 (33.5)	170 (28.5)	1.26 (0.87, 1.83)	.212	1.35 (0.87, 2.06)	.175
Hypoglycemia, *n* (%)	19 (11.8)	70 (11.7)	1.01 (0.57, 1.70)	.979	0.86 (0.46, 1.54)	.616
Severe hypoglycemia, *n* (%)	6 (3.7)	13 (2.2)	1.74 (0.60, 4.48)	.270	1.36 (0.43, 3.92)	.581
Any medical intervention for hypoglycemia within 48 h, *n* (%)	24 (14.9)	89 (14.9)	1.00 (0.60, 1.61)	>.999	0.83 (0.46, 1.46)	.526
Dextrose gel within 48 h, *n* (%)	22 (13.7)	82 (13.7)	0.99 (0.59, 1.62)	.982	0.83 (0.45, 1.48)	.542
D10 bolus within 48 h, *n* (%)	3 (1.9)	11 (1.8)	1.01 (0.18, 3.89)	>.999	–	–

Abbreviations: BMZ, betamethasone; D10, 10% dextrose solution; OR, odds ratio; CI, confidence interval.

a To investigate the association between timing from last betamethasone dose to delivery, unadjusted logistic regression models were used. Odds ratio and 95% confidence intervals were obtained. Fisher’s exact test was used for D10 bolus within 48 h due to small cell counts.

b Adjusted logistic regression models were used to adjust for diabetes, hypertension, birthweight category, and indication for antenatal steroids.

c Recent BMZ defined as last dose of betamethasone administration <14 days prior to delivery.

d Remote BMZ defined as last dose of betamethasone administration >14 days prior to delivery.

## Data Availability

The data that support the findings of this study are available from the corresponding author, LAB, upon request.
