# Supplementary material for: Betamethasone latency period and neonatal hypoglycemia in term infants
Source: J Matern Fetal Neonatal Med. Author manuscript; Available in PMC 2025 Sep 12. (PMC12426955; doi:10.1080/14767058.2025.2540477)
Supplement: Supplemental Table 1 [file NIHMS2102316-supplement-Supplemental_Table_1.docx]

**Supplemental Table 1.** Comparison of glucose and hypoglycemia outcomes for last BMZ dose < 3 days versus >14 days prior to delivery.

| **Variable** | **BMZ < 3 days^a^ (N=37)** | **BMZ > 14 days^b^ (N=597)** | **P-value** |
| --- | --- | --- | --- |
| Any glucose measurement within 48 hours, n (%) | 15 (40.5) | 170 (28.5) | 0.117 |
| Hypoglycemia (glucose < 40 mg/dL), n (%) | 5 (13.5) | 70 (11.7) | 0.792 |
| Severe hypoglycemia (glucose < 25 mg/dL), n (%) | 1 (2.7) | 13 (2.2) | 0.573 |
| Any medical intervention for hypoglycemia within 48 hours, n (%) | 6 (16.2) | 89 (14.9) | 0.829 |
| Dextrose gel within 48 hours, n (%) | 5 (13.5) | 82 (13.7) | 0.970 |
| D10 bolus within 48 hours, n (%) | 1 (2.7) | 11 (1.8) | 0.517 |

^a^ Defined as last dose of BMZ administered <3 days prior to delivery.

^b^ Defined as last dose of BMZ administered >14 days prior to delivery.

^c^ P-value is for Chi-square or Fisher’s exact test.

GA *gestational age. BMZ* betamethasone. *D10* 10% dextrose.
